# CircMRPS35 suppresses gastric cancer progression via recruiting KAT7 to govern histone modification

**DOI:** 10.1186/s12943-020-01160-2

**Published:** 2020-03-12

**Authors:** Mengmeng Jie, Yaran Wu, Mengyuan Gao, Xinzhe Li, Cheng Liu, Qin Ouyang, Qingyun Tang, Changyu Shan, Yangfan Lv, Kebin Zhang, Qian Dai, Yang Chen, Shuo Zeng, Chenglin Li, Liting Wang, Fengtian He, Changjiang Hu, Shiming Yang

**Affiliations:** 1 Department of Gastroenterology, Xinqiao Hospital, Third Military Medical University, Chongqing, 400037 China; 2 Department of Medicinal Chemistry, College of Pharmacy, Third Military Medical University, Chongqing, 400038 China; 3 Department of Pathology, Xinqiao Hospital, Third Military Medical University, Chongqing, 400037 China; 4 Central Laboratory, Xinqiao Hospital, Third Military Medical University, Chongqing, 400037 China; 5 Biomedical Analysis Center, Third Military Medical University, Chongqing, 400038 China; 6 Department of Biochemistry and Molecular Biology, College of Basic Medical Sciences, Third Military Medical University, Chongqing, 400038 China

**Keywords:** Circular RNA, Gastric cancer, Histone modification, Acetylation

## Abstract

**Background:**

Aberrant expression of circular RNAs contributes to the initiation and progression of cancers, but the underlying mechanism remains elusive.

**Methods:**

RNA-seq and qRT-PCR were performed to screen differential expressed circRNAs between gastric cancer tissues and adjacent normal tissues. Candidate circRNA (circMRPS35) was screened out and validated by qRT-PCR. Cell proliferation and invasion ability were determined by CCK-8 and cell invasion assays. RNA-seq, GO-pathway, RNA pull-down and ChIRP were further applied to search for detailed mechanism.

**Results:**

Here, a novel circRNA named circMRPS35, was screened out by RNA-seq in gastric cancer tissues, whose expression is related to clinicopathological characteristics and prognosis in gastric cancer patients. Biologically, circMRPS35 suppresses the proliferation and invasion of gastric cancer cells in vitro and in vivo. Mechanistically, circMRPS35 acts as a modular scaffold to recruit histone acetyltransferase KAT7 to the promoters of FOXO1 and FOXO3a genes, which elicits acetylation of H4K5 in their promoters. Particularly, circMRPS35 specifically binds to FOXO1/3a promoter regions directly. Thus, it dramatically activates the transcription of FOXO1/3a and triggers subsequent response of their downstream target genes expression, including p21, p27, Twist1 and E-cadherin, resulting in the inhibition of cell proliferation and invasion. Moreover, circMRPS35 expression positively correlates with that of FOXO1/3a in gastric cancer tissues.

**Conclusions:**

Our findings not only reveal the pivotal roles of circMRPS35 in governing histone modification in anticancer treatment, but also advocate for triggering circMRPS35/KAT7/FOXO1/3a pathway to combat gastric cancer.

## Background

Gastric cancer is one of the most common malignancy and it has become the third leading cause of cancer deaths in China [1]. Although comprehensive therapies, including surgery, radiotherapy, chemotherapy and biological treatment, have been adopted, the 5-year survival rate is still quite poor, partially due to the lack of knowledge on mechanisms of gastric cancer progression [2, 3]. To this end, more effective biomarkers and targets urgently needed to be discovered for better diagnosis and treatment of gastric cancer.

Circular RNAs (circRNAs) are a novel class of noncoding RNAs that are characterized by a covalent closed loop structure [4, 5]. Although circRNAs were first detected more than 20 years ago, their functions have not been explored until recently [6–8]. With the development of high-throughput sequencing and bioinformatics analysis, large numbers of circRNAs have been successfully identified and they are abundant, conserved, tissue-specific and development-specific in mammalian cells [9, 10]. Unlike their linear counterparts, circRNAs contain no 5′ to 3′ polarity or polyadenylation tails. CircRNAs are resistant to the digestion of RNase R and are highly stable in vivo compared with their parent genes. It is well established that inverted repeated Alu elements, exon skipping and RNA binding proteins facilitate and regulate the formation of circRNAs [11–13].

It has been characterized that circRNAs are widely involved in physiological and pathological processes, such as diabetes [14], neurological disorders [4] and especially cancer [15]. CircRNA BCRC-3 suppresses bladder cancer proliferation by acting as an endogenous miR-182-5p sponge, which results in the upregulation of p27 [16]. In non-small cell lung cancer, circPTK2 inhibits TGF-β-mediated epithelial mesenchymal transition and metastasis through sponging miR-429/miR-200b-3p and regulating target gene TIF1γ expression [17]. In summary, circRNAs may serve as potential diagnostic biomarkers of cancer and represent novel therapeutic targets. Given the tight relationship between circRNAs and tumorigenensis and progression, it is worthwhile to investigate the functions and underlying mechanisms of circRNAs in gastric cancer.

In this study, genome-wide circRNAs expression profiles were initially examined in gastric tumors and paired adjacent normal tissues, and a novel circRNA (hsa_circ_0000384) derived from MRPS35 gene, named circMRPS35, was characterized as a tumor suppressor gene in gastric cancer. Notably, in vitro and in vivo experiments revealed that circMRPS35 strongly suppressed the proliferation and invasion of gastric cancer. Mechanistically, mass spectrometry combined with RNA-seq revealed that circMRPS35 recruited KAT7 and increased H4K5 acetylation levels in FOXO1 and FOXO3a promoter regions, which altered their downstream genes expression, including p21, p27, Twist1 and E-cadherin, resulting in the inhibition of cell proliferation and invasion. Particularly, circMRPS35 specifically binds to FOXO1/3a promoter regions directly. Taken together, the novel circMRPS35 governing histone modification for FOXO1/3a activation, may serve as a promising diagnostic marker and therapeutic target to combat gastric cancer.

## Materials and methods

### Human subjects

All gastric cancer tissues and paired adjacent tissues for RNA-seq and qRT-PCR validation were collected from the Department of General Surgery of the Southwest and Xinqiao Hospital, Third Military Medical University. All tissues were immediately preserved in liquid nitrogen. Written informed consent was obtained from all patients and the study was approved by the Ethics Committee of the Second Affiliated Hospital of Third Military Medical University (ChiCTR1900026337). The human tissue microarrays containing 160 pairs of human gastric cancer tissues and corresponding adjacent noncancerous tissues were purchased from Shanghai Outdo Biotech. Co. Ltd. (Shanghai, China) (Additional file 1: Table S1).

### Culture and maintenance of cell lines

AGS, HGC27 and GES-1 cell lines were obtained from American Type Culture Collection (Manassas, Virginia, USA), MKN45, MKN74 and MKN28 were from JCRB Cell Bank (National Institute of Hygienic Sciences, Tokyo), BGC823 and SGC7901 cell lines were purchased from Cell Bank of the Shanghai Institute for Biological Sciences (Chinese Academy of Sciences, Shanghai, China), and MGC803 was from National Infrastructure of Cell Line Resource (Beijing, China). All cell lines were genotyped for identity by Shanghai Biowing Applied Biotechnology Co., Ltd. and tested routinely for Mycoplasma contamination. They were cultured in Dulbecco’s modified Eagle’s medium supplemented with 10% fetal bovine serum and 1% penicillin-streptomycin (100 U/mL) at 37 °C in an atmosphere of 5% CO_2_. Genetic transcription was blocked by adding 2 μg/ml Actinomycin D (Cayman Chemical, Ann Arbor, Michigan) or DMSO as a control to the cell culture medium.

### RNA extraction and qRT-PCR

Total RNA was isolated by RNAiso Plus reagent (Takara, Otsu, Shiga, Japan) following the manufacturer’s protocol. One microgram of total RNA was reverse transcribed to cDNA using the PrimeScript RT Reagent Kit (Takara, Otsu, Shiga, Japan). The levels of circRNA and mRNA expression were measured by RT-PCR using SYBR Premix Ex Taq II (Takara) with the ABI 7500 StepOnePlus system (Applied Biosystems, CA, USA). β-actin was used as an internal control and each reaction was performed in triplicate. The primers are listed in Additional file 2: Table S2.

### RNase R treatment

MKN45 total RNA (10 μg) was incubated for 15 min at 37 °C with or without 3 U/μg RNase R (Epicentre Technologies, Madison, WI, USA), and RNA was subsequently purified through phenol-chloroform extraction as described previously [18].

### Population doubling time assay

The gastric cancer cell lines (SGC7901, MKN28, MKN74, AGS, BGC823, HGC27, MKN45 and MGC803) and GES-1 cells were seeded in 24-well plates at the density of 10^4^ cells per well. Cell counting was performed at the indicated time points. The cell growth curve was drawn by culture time as X-axis and cell number as Y-axis. Then the population doubling time was calculated according to the Patterson formula: Td = Tlg2 / lg (N_t_/N_0_), Td: population doubling time (h), T: time for cell number increasing from N_0_ to N_t_, N_0_: initial cell number, N_t_: cell number at day t.

### Transfection and Lentivirus package

DNA sequence of 4 exons of MRPS35 gene (exon 2 to exon 5), together with 728 bp upstream and 1062 bp downstream to the nonlinear splice sites, were synthesized. The fragment was inserted into pCDH-CMV-MCS-EF1-GFP+ Puro (CD513B-1) vector (Geenseed Biotech, Guangzhou, China) with BamHI and NotI restriction sites. The result of vector construction was verified by direct sequencing. For the transient transfection, the overexpression plasmid pCDH-CMV-CircMRPS35 and the corresponding control plasmid were transfected into gastric cancer cell lines using lipofectamine 3000 (Invitrogen) reverse transfection protocol according to the manufacturer’s instructions. For the lentivirus package, HEK-293 T cells were transfected with the core plasmid pLCDH-ciR (Geneseed Biotech, Guangzhou, China), with the psAX2 packaging plasmid and pMD2G envelope plasmid for 48 h to obtain the lentivirus supernatant. The lentivirus were named as Lv-Control and Lv-Circ. The shRNAs targeting circMRPS35 or random sequence were synthesized and inserted into lentivirial vector pGV248 (hU6-MCS-Ubiquitin-EGFP-IRES-puromycin) with AgeI and EcoR I (GeneChem, China). HEK-293 T cells were transfected with pGV248, together with the pHelper 1.0 and pHelper 2.0 packaging plasmid. The lentivirus were named as shNC, shCirc-1 and shCirc-2, respectively. For the construction of promoter activity reporter vectors, the promoter regions of FOXO1 and FOXO3a were cloned into the pGL3-Basic plasmid and were named pGL3-FOXO1 and pGL3-FOXO3a, respectively.

### Super-resolution microscopy

IF staining was performed followed by FISH. During IF staining, the sections were incubated with anti-KAT7 (mouse monoclonal, 1:200) and anti-H4K5ac (rabbit monoclonal, 1:200) overnight at 4 °C. Finally, the sections were stained with Alexa Fluor 647 donkey anti-mouse IgG (1:200, Invitrogen) and Alexa Fluor 488 goat anti-rabbit IgG (1:200) for 1 h at 37 °C. All fluorescent images were acquired using the super-resolution microscopy. Super-resolution microscopy was performed on a DeltaVision OMX V4 Blaze (GE Healthcare) as described previously [19]. In general, pixel registration was adjusted to be less than 1 pixel for all channels with 100 nm Tetraspeck beads (Molecular Probes).

### Immunohistochemical analysis and scoring

We quantitatively scored tissue sections using ImagePro Plus (Media Cybernetics, USA) as previously described [20]. The mean optical density of the selected area was determined by the software and represented the expression level of the candidates within tissues.

### Luciferase assay

The promoter activity reporter vectors (pGL3-FOXO1 and pGL3-FOXO3a) and pRL-TK, together with the circMRPS35 overexpression plasmid or si-Circ, were transfected into SGC7901 or MGC803 cells. The luciferase activity was measured with the luciferase assay substrate (Promega, Madison, WI) after 24 h. The transfection experiments were performed three times in triplicate, and the luciferase activity was normalized to the pRL-TK activity.

### RNA pull-down assay

RNA pull-down assays were carried out as described [21]. The biotin-labeled circMRPS35 or random oligo probe was incubated with streptavidin magnetic beads at room temperature for 1 h. Then the gastric cancer cell lysates were incubated with probe-beads complex at 4 °C overnight for the binding of RNA-associated proteins to RNA. Subsequently, the RNA-protein complexes were washed three times and eluted from beads. The eluted proteins were finally analyzed by mass spectrometry or Western blot.

### RNA binding protein Immunoprecipitation assay

RIP assays were carried out using the Magna RIP RNA-Binding Protein Immunoprecipitation Kit (Millipore, Bedford, MA) according to the manufacturer’s instructions. Cells were washed with ice-cold PBS (137 mM NaCl, 3 mM KCl, 8 mM Na_2_HPO_4_ and 1.5 mM KH_2_PO_4_, pH 7.4) and lysed in complete RIP lysis buffer (Millipore). Five micrograms of primary antibody (control rabbit IgG, KAT7, H4K5ac, BTF3 and TAF15) was incubated with magnetic beads for 30 min at room temperature. Cell lysates were incubated with beads-antibody complex at 4 °C overnight. The beads were washed 6 times with ice-cold RIP wash buffer and resuspended in proteinase K buffer. The immunoprecipitated RNA was purified by phenol-chloroform extraction and ethanol precipitation. qRT-PCR analysis was performed for downstream RNA detection.

### Chromatin Immunoprecipitation assay

Chromatin immunoprecipitation (ChIP) was performed using a ChIP assay kit (Thermo Fisher Scientific, Rockford, IL) according to the manufacturer’s instructions as described as previously [22]. Briefly, cells were crosslinked and the chromatin was sheared by enzymatic digestion with micrococcal nuclease. The digested chromatin was incubated with 5 μg H4K5ac, H4K12ac, H3K14ac antibody and IgG overnight at 4 °C, and 20 μl protein A/G plus agarose for 1 h at 4 °C. After IP elution and DNA recovery, the purified DNA was subjected to qPCR detection.

### Chromatin isolation by RNA purification (ChIRP)

CircMRPS35 antisense probe was designed at the back-spliced site (GTTCTTTCCGTCTTAAGACT). All probes were synthesized with BiotinTEG at the 3′ end. MGC803 cells were harvested for ChIRP assay as previously described [23].

### Molecular docking

The crystal structures of KAT7 and H4K5ac were retrieved from the Protein Data Bank (http://www.pdb.org/pdb/) and prepared by SYBYL-X 2.0 (including residue repair and energy minimization). The binding modes of circMRPS35 RNA fragment (AAGACGGA) with KAT7 were predicted by Surflex-Dock in SYBYL2.0. In the process of molecular docking, the original ligands were extracted out of the protein and generated banding pocket. Subsequently, RNA fragment was docked into the pocket. The docking complex of RNA fragment, KAT7 and H4K5ac was generated by ZDOCK in Discovery Studio by docking H4K5ac with KAT7/RNA complex.

### Statistical analysis

**Fig. 1:**
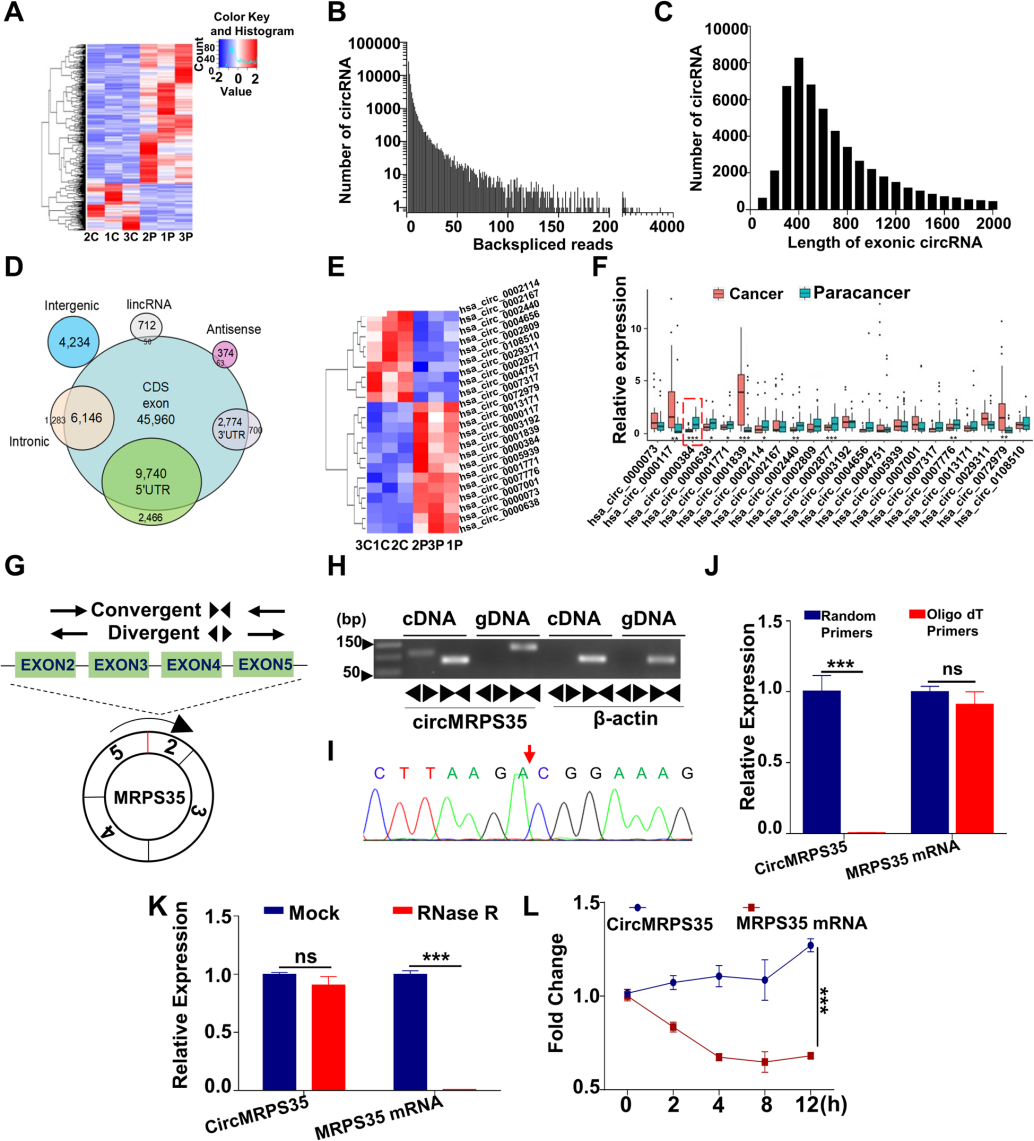
Identification and Validation of a Novel Circular RNA (circMRPS35) in Human Gastric Cancer Tissues. **a** Heat map of all differentially expressed circRNAs. Red represents upregulated circRNAs, and green represents downregulated circRNAs in gastric cancer tissues compared to the paracancer tissues. **b** The back-spliced reads distribution of identified circRNAs in three paired gastric cancer tissues and adjacent normal tissues. **c** The length distribution of the identified exonic circRNAs. **d** Genomic origin of the above circRNAs. **e** Heat map of the significantly differentially expressed circRNAs from **a**. **f** The differentially expressed circRNAs validated in 30 pairs of gastric cancer tissues and adjacent normal tissues by qRT-PCR assay. **g** Schematic diagram of circMRPS35 and the design of PCR primers. **h** PCR validation of the circMRPS35 amplified by divergent primers and convergent primers using the template cDNA and genomic DNA (gDNA) derived from MKN45 cells. β-actin, linear control. **i** The Sanger sequencing of the back-splice sites of the products from **h**. The red arrow represents the “head-to-tail” splicing sites of circMRPS35. **j** qRT-PCR analysis for the circMRPS35 and MRPS35 mRNA using the template cDNA reverse-transcribed by random primers and oligo dT primers. **k** qRT-PCR assay for the expression of circMRPS35 and MRPS35 mRNA in MKN45 cells treated with RNase R. **l** qRT-PCR assay for the expression of circMRPS35 and MRPS35 mRNA in MKN45 cells treated with the transcription inhibitor Actinomycin D (2 μg/ml) at the indicated time points. *P < 0.05, **P < 0.01, ***P < 0.001

The expression of circRNAs between 30 pairs of gastric cancer and adjacent normal cancer tissues were analyzed by ggplot2. The correlation coefficients were calculated by Pearson’s rank correlation test. The Kaplan-Meier method was used for survival analysis. For comparisons between two groups, student’s t test was applied if no significantly different variances existed. When more than two groups were compared, one-way ANOVA was performed followed by Tukey’s test. To calculate the *P* value between groups in Fig. 1, 3, 5l, c, h, i SFig 2F and 3D, two-way analysis of variance (ANOVA) analysis was performed with Prism 8. *P < 0.05, **P < 0.01, ***P < 0.001, ns = not significant. *P* < 0.05 was considered statistically significant.

## Results

### Differential expression profiles of circRNAs in human gastric cancer and adjacent normal tissues

RNA-seq of ribosomal RNA-depleted total RNA was used to profile circRNA expression in three paired gastric cancer tissues and adjacent normal tissues (Fig. 1a), and the raw data was accessible via GSE121445. A total of 57,623 distinct circRNAs were identified in these samples, and 35,120 of these circRNAs contained at least two independent back-spliced reads in at least two samples (Fig. 1b and Additional file 3: Table S3). Compared with circBase [24], there were 29,007 matched circRNAs and 28,616 novel circRNAs in our study (Additional file 4: Table S4). The majority of exonic circRNAs were less than 1500 nucleotides (nt) and the median length was ~ 500 nt (Fig. 1c and Additional file 5: Table S5). We next annotated these identified circRNA candidates using the RefSeq database. Most of these circRNAs originated from exons, and others aligned with introns, 3′-UTR, 5′-UTR, intergenic region, and antisense sequences, etc. (Fig. 1d). Thirteen candidate circRNAs were significantly downregulated and 9 were upregulated in gastric cancer tissues compared with adjacent normal tissues (Fig. 1e). The expression of the candidate circRNAs was further validated in 30 pairs of gastric cancer and adjacent normal tissues by quantitative real time polymerase chain reaction (qRT-PCR) assay. Figure 1f and Additional file 6: Fig. S1 show that 9 circRNAs exhibited statistically significant differences, among which the *p* value of hsa_circ_0000384, derived from the MRPS35 gene exon 2, 3, 4 and 5 (termed circMRPS35), was the smallest.

### Characterization of circMRPS35 in gastric cancer cells

To further clarify the characteristics of the novel circular RNA circMRPS35, divergent and convergent primers were designed to amplify the back-spliced and linear products, respectively (Fig. 1g). Figure 1h clearly reveals that divergent primers amplified circMRPS35 in cDNA but not in gDNA. The head-to-tail splicing of exon 2 and exon 5 was confirmed by Sanger sequencing (Fig. 1i). In view of the fact that circRNAs have no poly-A tail, random and oligo dT primers were applied in the reverse transcription PCR (RT-PCR). Figure 1j shows that circMRPS35 reverse-transcribed by the Oligo dT primers was much less than that by random primers. Figure 1k shows that circMRPS35 is much more resistant to RNase R digestion compared to the linear mRNA of MRPS35. Figure 1l demonstrated that circMRPS35 was dramatically more stable than MRPS35 mRNA after the transcription inhibitor Actinomycin D treatment in MKN45 cells. Collectively, circMRPS35 is a stable circRNA with low expression in gastric cancer tissues.

### CircMRPS35 expression correlates with good prognosis in gastric cancer patients

**Fig. 2:**
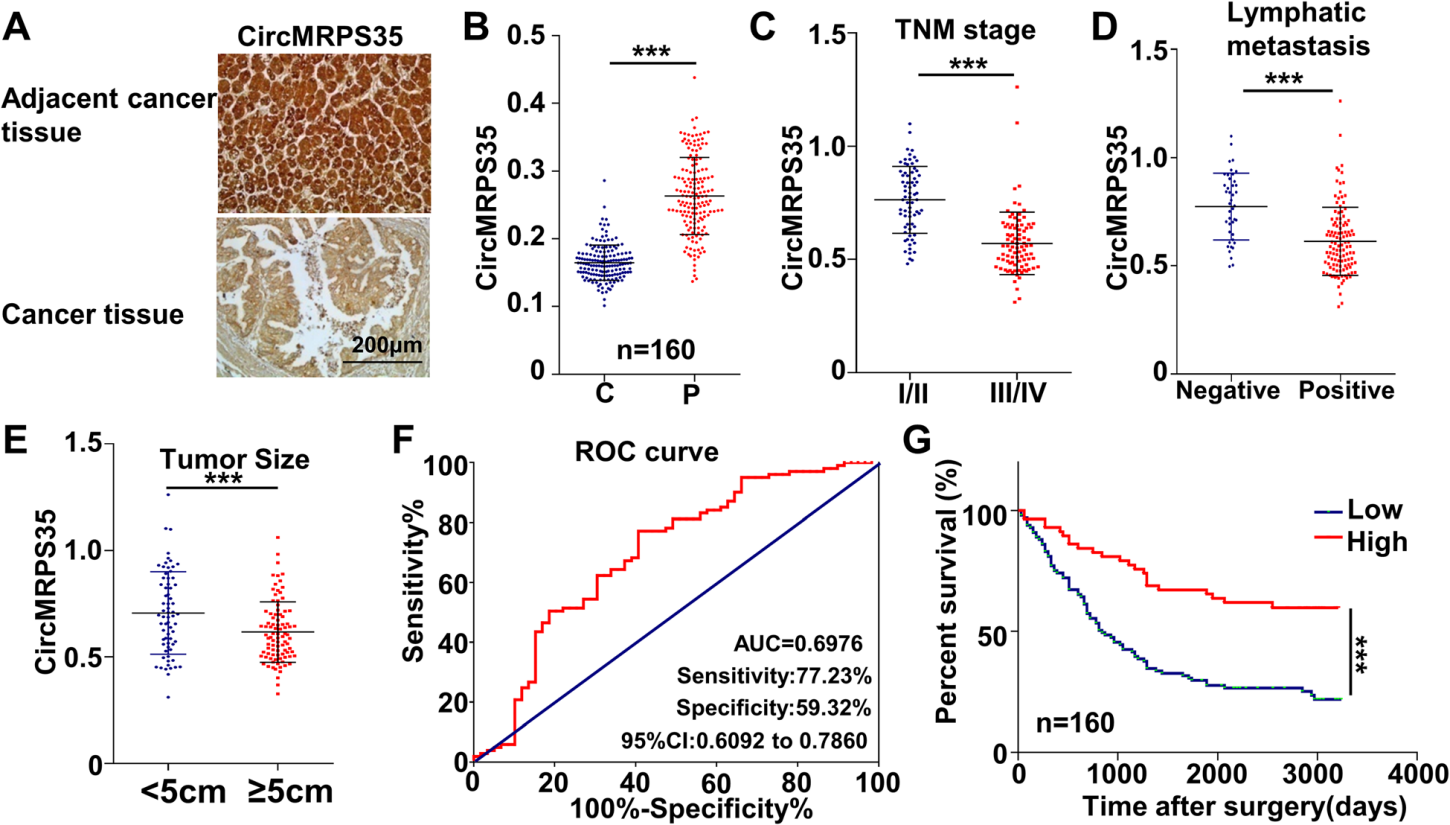
CircMRPS35 Expression is correlated with Good Prognosis of Gastric Cancer Patients. **a** Representative images of circMRPS35 in gastric cancer tissues and corresponding adjacent tissues through ISH. **b** ISH assay for circMRPS35 expression in 160 pairs of gastric cancer and adjacent tissues. **c-e** The association between circMRPS35 expression and TNM stage **c**, lymphatic metastasis **d**, tumor size **e** in gastric cancer patients (*P* < 0.01). **f** The ROC curve for predicting patient survival time using circMRPS35 expression. **g** Kaplan–Meier analysis of overall survival according to circMRPS35 expression level (*P* < 0.01)

To evaluate the association between circMRPS35 expression and the clinicopathological features, in situ hybridization (ISH) was performed in 160 pairs of gastric cancer and adjacent tissues. The expression of circMRPS35 was significantly decreased in gastric cancer tissues compared with adjacent tissues (Fig. 2a and b). The expression level of circMRPS35 was negatively correlated with advanced tumor node metastases (TNM) stage, lymphatic metastasis and tumor size (Fig. 2c-e). The receiver operating characteristic (ROC) curve analysis demonstrated that the area under the curve of circMRPS35-based prediction was 0.6976, indicating that circMRPS35 could be applied for the prediction of patients’ prognosis (Fig. 2f). To pinpoint the best cut-off value after the generation of the ROC curve, the balance between the sensitivity and specificity was found. To maximize both of sensitivity and specificity, the Youden’s index was introduced, which was Maximum = Sensitivity + Specificity - 1. From the ROC curve, we found the Youden’s index was maximal when sensitivity was 77.23% and specificity was 59.32%. Thus the value of the circMRPS35 expression at this point is the best cut-off value to determine the high or low expression levels. Figure 2g clearly shows that the lower expression of circMRPS35 was strongly associated with a shorter survival time of patients with gastric cancer. Thus, circMRPS35 is obviously clinically relevant in gastric cancer.

### CircMRPS35 inhibits gastric cancer cells proliferation and metastasis in vitro

**Fig. 3:**
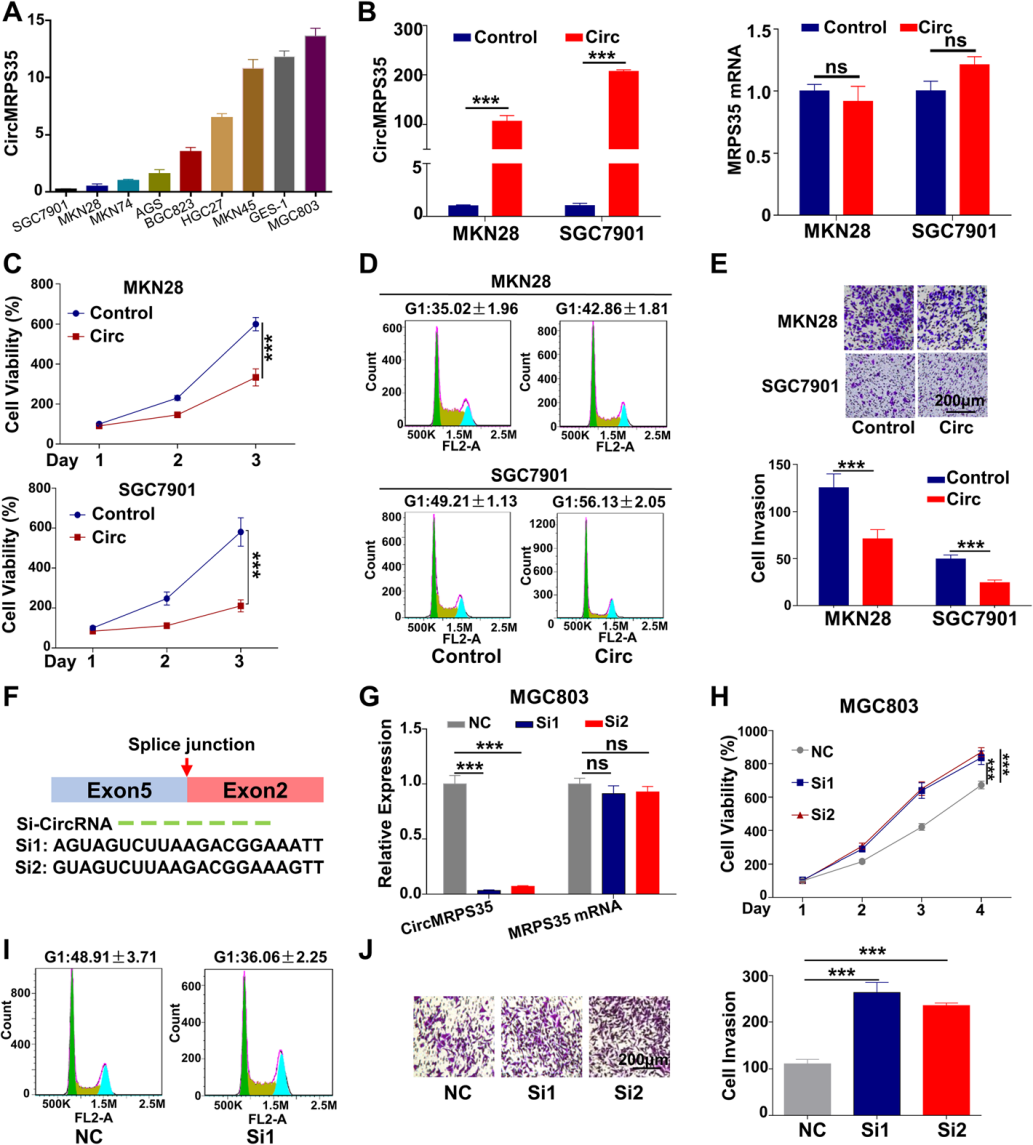
CircMRPS35 Inhibits Gastric Cancer Cells Proliferation and Metastasis in vitro**. a** qRT-PCR assay for circMRPS35 expression in different human gastric cancer cell lines and GES-1. **b** qRT-PCR analysis of circMRPS35 and MRPS35 mRNA after the transfection of overexpression plasmids into SGC7901 and MKN28 cells for 24 h. (**c**) CCK-8 analysis of the above cells in **b** on the indicated days. **d** Flow cytometry of the cell cycle of the above cells in **b**. **e** Cell invasion assay after overexpression of circMRPS35. Six hours after the transfection of overexpression plasmid, SGC7901 and MKN28 cells were resuspended and seeded in transwells for another 48 h, and the number of invasion cells was calculated. **f** Schematic illustration of two siRNAs targeting the back-splice junction of circMRPS35 (Si1 and Si2). **g** qRT-PCR assay of interfering efficacy after transfection of the siRNAs into MGC803 cells for 24 h. **h-j** CCK-8 **h**, flow cytometry **i** and cell invasion assay **j** of the above cells in **g**. *P < 0.05, **P < 0.01, ***P < 0.001

To evaluate the function of circMRPS35, qRT-PCR assay was applied to examine the expression of circMRPS35 in various human gastric cancer cell lines and GES-1 cells. Figure 3a shows that circMRPS35 level was relatively low in SGC7901 and MKN28 cells, and relatively high in MGC803 and MKN45 cells. Moreover, the expression of circMRPS35 was positively correlated with the doubling time of the gastric cancer cells (Additional file 7: SFig 2a), suggesting its potential tumor suppressor role in cancer. CircMRPS35 was obviously increased after the transfection of its overexpression plasmid pCDH-CMV-CircMRPS35 in SGC7901 and MKN28 cells compared with the pCDH-CMV-Control vector, while the MRPS35 mRNA and protein expression were not changed obviously (Fig. 3b and Additional file 7: SFig 2b and 2c). Figure 3c and d show that circMRPS35 significantly suppressed cell growth and induced cell cycle arrest at G1 phase. Figure 3e further reveals that it inhibited the invasion of gastric cancer cells. Moreover, siRNAs targeting the back-splice sites of circMRPS35 were designed (Fig. 3f) and these siRNAs significantly decreased circMRPS35 expression without any effect on MRPS35 mRNA and protein expression (Fig. 3g and Additional file 7: SFig 2d and 2e). In addition, knockdown of circMRPS35 markedly promoted cell proliferation, cell cycle acceleration and invasion of MGC803 (Fig. 3h-j) and MKN45 cells (Additional file 7: SFig 2f-h). These results definitely verify that circMRPS35 modulates the behaviors of gastric cancer cells in vitro.

### CircMRPS35 suppresses the growth and metastasis of gastric cancer cells in vivo

**Fig. 4:**
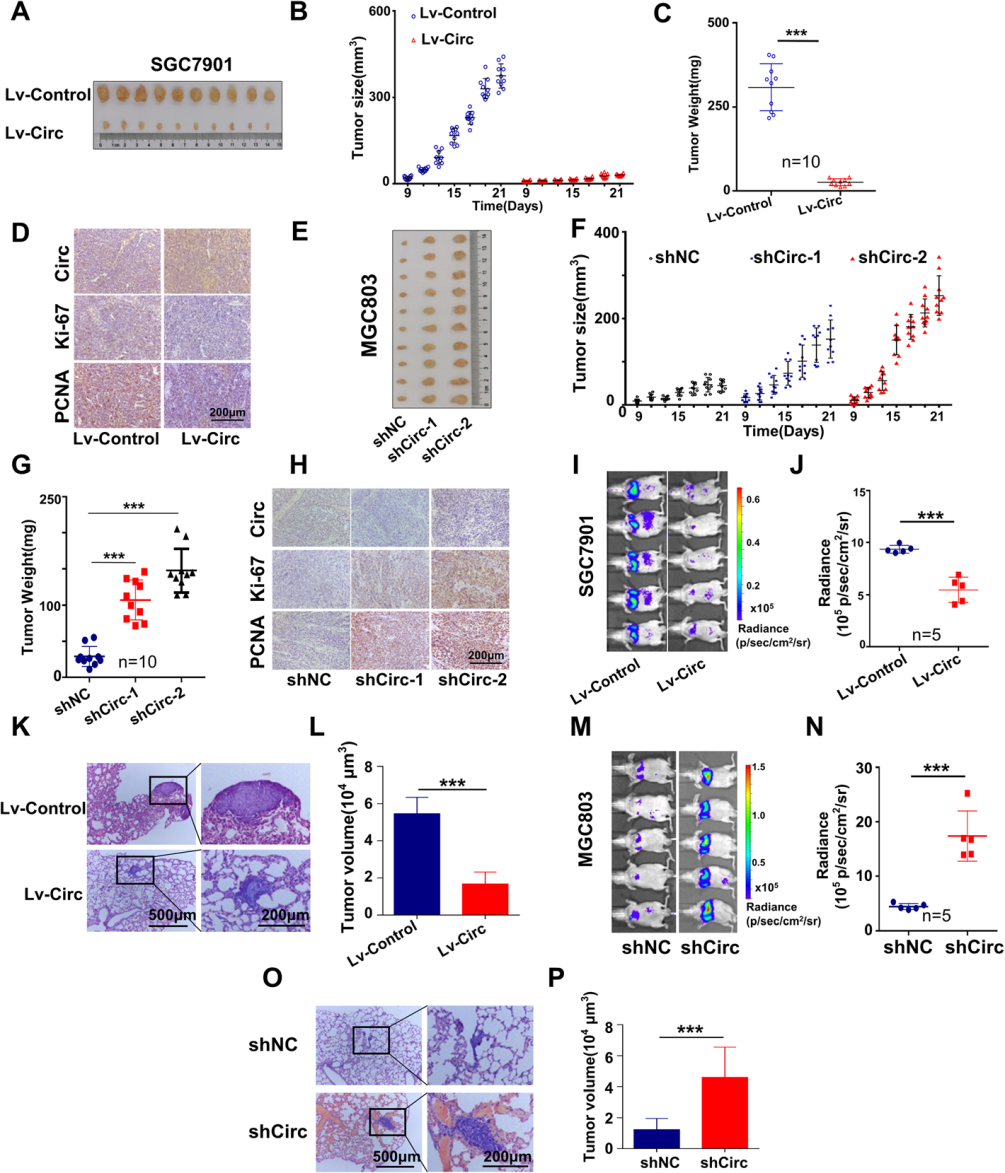
Enforced Expression of CircMRPS35 Suppresses the Growth and Metastasis of Gastric Cancer Cells in vivo. **a** The image of tumors in nude mice bearing gastric cancer cells. The subcutaneous transplanted model of human gastric cancer was established with SGC7901 cells infected with Lv-circMRPS35 (Lv-Circ) or Lv-control in nude mice (*n* = 10 in each group). **b** Tumor volume measured on the indicated days. **c** Weight of tumor masses. All nude mice were sacrificed on the 21th day after inoculation, and the tumor masses were weighed. **d** Representative images of circMRPS35 stained by ISH and Ki-67, PCNA stained by IHC. Scale bar, 200 μm. **e** The image of tumors in nude mice bearing MGC803 cells infected with shCirc-1, shCirc-2 targeting circMRPS35 and shNC lentivirus. **f**-**h** Tumor size **f**, tumor weight **g** and staining of circMRPS35, Ki-67 and PCNA **h** in the tumors of the above model in **e**. **i** The image of in vivo bioluminescence imaging. Stable SGC7901 cells (infected with Lv-circMRPS35-luci (Lv-Circ) or Lv-control-luci (Lv-Control)) were injected into nude mice (*n* = 5 in each group) via the tail vein. The luminescence intensity of the mice was measured through in vivo small animal imaging technology 4 weeks later. **j** Statistical analysis of the luminescence intensity. **k** H&E staining of representative metastatic lesions in the lungs of nude mice. After the mice were sacrificed, the lungs were divided into eight parts on average, and each part was used for the calculation of metastatic lesion area. Scale bar, 500 μm (left), 200 μm (right). **l** The total area of metastatic lesions for each mice representing the metastatic tumor volume in the lungs. **m** The images of in vivo bioluminescence imaging. Stable MGC803 cells (shCirc and shNC infected with luciferase-expressing lentivirus) were injected into nude mice (*n* = 5 in each group) via the tail vein. The luminescence intensity of the mice was measured through in vivo small animal imaging technology 4 weeks later. **n** Statistical analysis of the luminescence intensity. **o** H&E staining of representative metastatic lesions in the lungs of nude mice. **p** The total area of metastatic lesions for each mice. *P < 0.05, **P < 0.01, ***P < 0.001

To further elucidate the role of circMRPS35 in vivo, stable gastric cancer cell lines with gain of function and loss of function of circMRPS35 were constructed. As shown in Fig. 4a-c, circMRPS35 decreased tumor growth and weight in xenograft mice models. Immunohistochemical staining (IHC) further illustrated that Ki-67 and PCNA were decreased in circMRPS35-overexpressed tumors (Fig. 4d). CircMRPS35 knockdown by shRNA lentivirus in MGC803 increased tumor growth, tumor weight and the expression of Ki-67 and PCNA (Fig. 4e-h). Moreover, live imaging systems for small animals and H&E staining showed that circMRPS35 remarkably reduced metastatic foci in the lungs of nude mice in vivo (Fig. 4i-l). Knockdown of circMRPS35 increased the number of metastatic foci in the lungs of nude mice (Fig. 4m-p). These results further validate that circMRPS35 inhibits the growth and metastasis of gastric cancer in vivo.

### CircMRPS35 inhibits gastric cancer progression through transcriptional activation of FOXO1 and FOXO3a

**Fig. 5:**
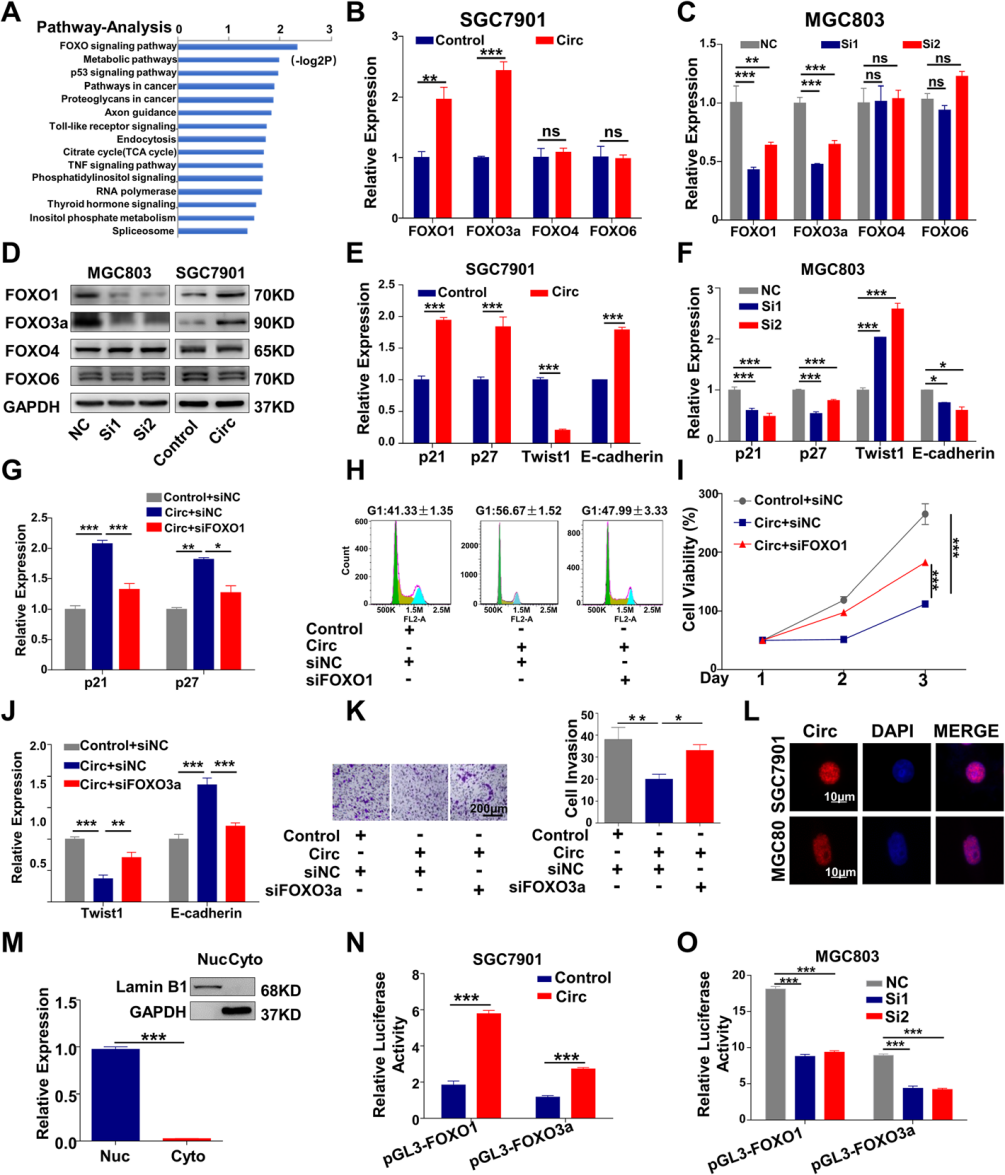
CircMRPS35 Inhibits Gastric Cancer Progression by Upregulating the Transcriptional Activity of FOXO1 and FOXO3a. **a** RNA-seq and pathway analysis of circMRPS35-mediated mRNA expression profiles. **b-c** qRT-PCR assay for FOXO1, FOXO3a, FOXO4 and FOXO6 mRNA after overexpression or knockdown of circMRPS35. SGC7901 cells were transfected with circMRPS35 overexpression plasmid **b**, while MGC803 cells were transfected with siRNAs targeting circMRPS35 and corresponding control for 24 h **c**. **d** Western blot for FOXO1, FOXO3a, FOXO4 and FOXO6 after overexpression or knockdown of circMRPS35. Cells were treated as in **b, c** and harvested 48 h later. **e**-**f** qRT-PCR assay for FOXO1 target genes p21 and p27, and FOXO3a target genes Twist1 and E-cadherin. Cells were treated as in **b, c** and harvested 48 h later. **g** p21 and p27 mRNA expression after FOXO1 knockdown in circMRPS35-overexpressed SGC7901 cells. SGC7901 cells were transfected with circMRPS35 overexpression plasmid and control plasmid, together with siRNA targeting FOXO1 and siNC for 48 h. **h** Flow cytometry in SGC7901 cells. Cells were treated as in **g**. **i** CCK-8 assay in SGC7901 cells. Cells were treated as in **g**, six hours after transfection, cells were resuspended and seeded in 96-well plates, and CCK-8 assays were performed on the indicated days. **j** Twist1 and E-cadherin mRNA expression after FOXO3a knockdown in circMRPS35-overexpressed SGC7901 cells. SGC7901 cells were transfected with circMRPS35 overexpression plasmid and control plasmid, together with siRNA targeting FOXO3a and siNC for 48 h. **k** Cell invasion assay was performed in the above cells as in **j** and the number of invasion cells was calculated. Scale bar, 200 μm. **l** FISH assay of circMRPS35 in both SGC7901 and MGC803 cells. Nuclei were stained with DAPI. Scale bar, 10 μm. **m** qRT-PCR analysis for circMRPS35 expression and Western blot for Lamin B1, GAPDH. The nuclear and cytoplasmic fractions of MGC803 cells were isolated, followed by qRT-PCR and Western blot assays. Lamin B1 as a nuclear control, and GAPDH as a cytoplasmic control. **n** The luciferase activity of FOXO1 and FOXO3a promoters after circMRPS35 overexpression. SGC7901 cells in 24-well plates were transfected with circMRPS35 plasmids or control, together with pGL3-basic-FOXO1 (pGL3-FOXO1) or pGL3-basic-FOXO3a (pGL3-FOXO3a) plasmid. The luciferase activity was measured 24 h later. **o** The luciferase activity of FOXO1 and FOXO3a reporters after circMRPS35 knockdown. MGC803 cells in 24-well plates were transfected with circMRPS35 siRNA, together with the luciferase reporters. The luciferase activity was measured 24 h later. *P < 0.05, **P < 0.01, ***P < 0.001

To explore the mechanism of circMRPS35 on the suppression of proliferation and invasion of gastric cancer cells, RNA-seq analysis was performed to elucidate the gene expression profiles after circMRPS35 knockdown (raw data accessible via GEO number GSE121382). GO enrichment and KEGG pathway analysis indicated that FOXO signaling is the most correlated pathway among the circMRPS35-regulated signaling involved in cancer (Fig. 5a). As the FOXO family is evolutionally conserved and consists of four members in mammals, FOXO1, FOXO3a, FOXO4 and FOXO6 [25], their expression were detected after exogenous expression of circMRPS35. Figure 5b-d show that circMRPS35 dramatically increased both the mRNA and protein levels of FOXO1 and FOXO3a without notable effects on FOXO4 and FOXO6. However, CircMRPS35 knockdown showed no obvious effect on phosphorylation of FOXO1 and FOXO3a (Additional file 8: SFig 3a). Previous studies have validated that the target genes of FOXO1 include p21 and p27 [26], while FOXO3a target genes include Twist1, E-cadherin, Snail and Y-box–binding protein 1 (YB-1) [27, 28]. Figure 5e-f show that circMRPS35 altered the expression of p21, p27, Twist1 and E-cadherin accordingly, without detectable effects on Snail and YB-1 (data not shown). Moreover, reduction of FOXO1 significantly attenuated circMRPS35-facilitated p21 and p27 expression, which rescued the cell cycle arrest and suppression of proliferation in SGC7901 cells (Fig. 5g-i). FOXO3a knockdown decreased circMRPS35-elicited E-cadherin expression, and rescued circMRPS35-induced suppression of Twist1 expression and cell invasion (Fig. 5j and k). Moreover, overexpression of FOXO1 and FOXO3a partially reversed the circMRPS35-altered target genes expression, cell proliferation and invasion in MGC803 cells (Additional file 8: SFig 3b-f). Fluorescent in situ hybridization (FISH) indicated that circMRPS35 was predominantly localized in the nucleus of SGC7901 and MGC803 cells (Fig. 5l), which was further confirmed by the separation analysis of the nuclear and cytoplasmic RNA (Fig. 5m). Dual luciferase reporter assay illustrated that circMRPS35 significantly enhanced the promoter activity of FOXO1 and FOXO3a and vice versa (Fig. 5n and o). In summary, circMRPS35 suppresses the behaviors of gastric cancer cells through transcriptional activation of FOXO1 and FOXO3a.

### CircMRPS35 expression parallels that of FOXO1 and FOXO3a in gastric cancer tissues

**Fig. 6:**
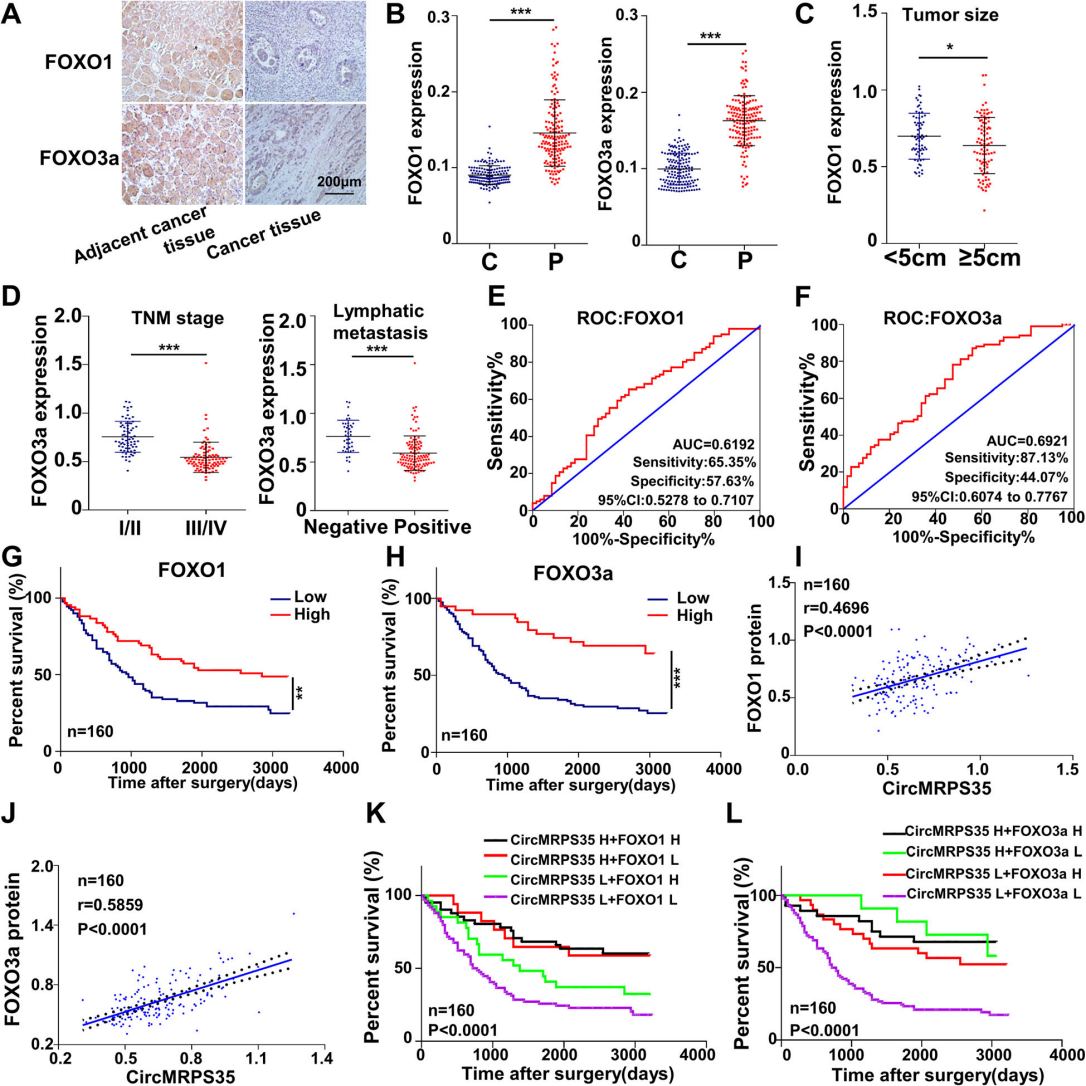
CircMRPS35 Expression Parallels that of FOXO1 and FOXO3a in Gastric Cancer Tissues. **a** Representative IHC staining of FOXO1 and FOXO3a in gastric cancer tissues and corresponding adjacent tissues. Scale bar, 200 μm. **b** FOXO1 and FOXO3a expression in 160 pairs of gastric cancer and adjacent tissues. **c** The association between FOXO1 expression and tumor size in gastric cancer patients (*P* < 0.05). **d** The association between FOXO3a expression and TNM stage/lymphatic metastasis in gastric cancer patients (*P* < 0.01). **e**-**f** The ROC curves for predicting patient survival time using FOXO1 **e** or FOXO3a **f** expression. **g**-**h** Kaplan–Meier analyses of overall survival according to FOXO1 **g** or FOXO3a **h** expression levels. **i**-**j** The correlations between the expression levels of circMRPS35 and FOXO1 **i** or FOXO3a **j** (*P* < 0.0001). **k** Kaplan–Meier analyses of overall survival time according to the combination of circMRPS35 and FOXO1. (*P* < 0.0001). **l** Kaplan–Meier analyses of overall survival time according to the combination of circMRPS35 and FOXO3a. (*P* < 0.0001)

We next investigated the clinical associations among circMRPS35, FOXO1 and FOXO3a in gastric cancer tissues. Either FOXO1 or FOXO3a expression was significantly decreased in gastric cancer tissues (Fig. 6a and b). The expression level of FOXO1 was negatively associated with tumor size, and FOXO3a expression was negatively associated with advanced TNM stage and lymphatic metastasis (Fig. 6c and d). The ROC curves indicated that both FOXO1 and FOXO3a expression could be applied for the prediction of patient prognosis (Fig. 6e and f). The low expression of FOXO1 or FOXO3a was strongly associated with poor survival of gastric cancer patients (Fig. 6g and h). Moreover, FOXO1 and FOXO3a expression were positively correlated with that of circMRPS35 (Fig. 6i and j). Interestingly, the shortest survival time was observed in the groups with low expression of circMRPS35 and FOXO1 or FOXO3a (Fig. 6k and l). Collectively, these data strongly indicate that circMRPS35-mediated FOXO1 and FOXO3a signaling plays a crucial role in gastric cancer.

### CircMRPS35 acts as a modular scaffold to recruit KAT7 to the promoters of FOXO1 and FOXO3a gene

**Fig. 7:**
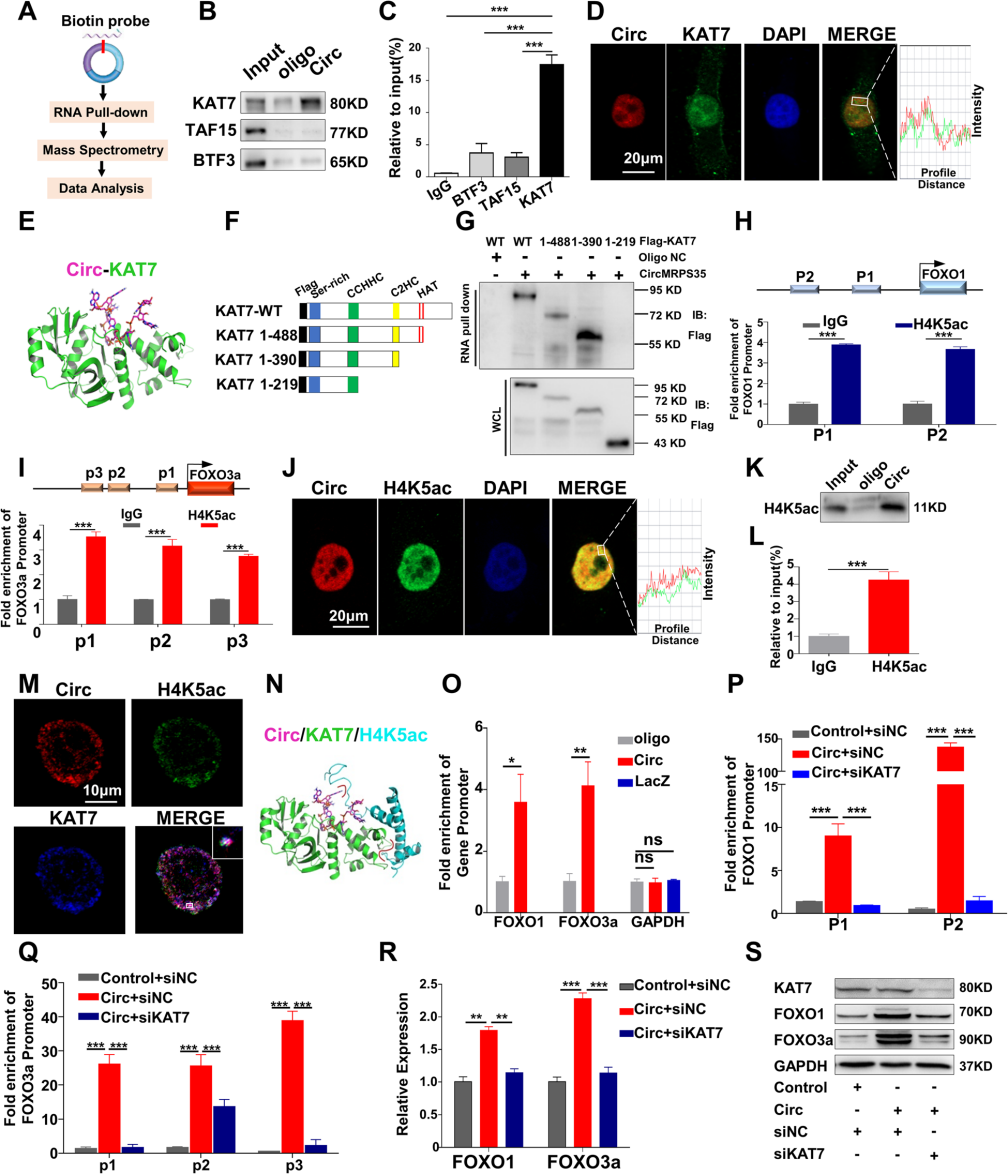
CircMRPS35 Acts as a Modular Scaffold to Recruit KAT7 to the Promoters of FOXO1 and FOXO3a Gene. **a** The experimental design for pull-down assay. RNA pull-down was performed using biotinylated circMRPS35 probe, followed by mass spectrometry. **b** RNA pull-down assay followed by western blot for candidate proteins KAT7, TAF15 and BTF3 in MGC803 cells. **c** RIP assay for the binding of three candidate proteins with circMRPS35. RIP was performed using KAT7, TAF15 and BTF3 antibodies, followed by qRT-PCR assay for circMRPS35 expression in MGC803 cells. **d** FISH for circMRPS35 and IF for KAT7 in MGC803 cells. The profiles of colocalization were also provided. Scale bar, 20 μm. **e** The molecular docking of the interaction between circMRPS35 fragment (back-splice sites, sequence: AAGACGGA) (marked stick) and KAT7 (shown as green). **f** The design of the truncated KAT7 expression plasmids. **g** RNA pull-down assay after transfection of wild type and truncated KAT7 expression plasmids using biotin-labelled oligo or circMRPS35 probes in MGC803 cells. **h**-**i** ChIP assay for H4K5ac level in FOXO1 **h** and FOXO3a **i** promoter regions. Final DNA extractions were PCR amplified using primers that cover P1 (− 839~ − 1028), P2 (− 1865~ − 2045) in FOXO1 promoter and p1 (− 1560~ − 1739), p2 (− 1976~ − 2155), p3 (− 3159~ − 3347) in FOXO3a promoter. **j** FISH for circMRPS35 and IF for H4K5ac in MGC803 cells. The profiles of colocalization are also provided. Scale bar, 20 μm. **k** RNA pull-down assay followed by western blot for H4K5ac expression in MGC803 cells. **l** RIP assay for the binding of H4K5ac with circMRPS35. RIP was performed using H4K5ac antibody, followed by qRT-PCR for circMRPS35 expression in MGC803 cells. **m** Colocalization analysis of circMRPS35, KAT7 and H4K5ac by super high resolution microscopy in MGC803 cells. Scale bar, 10 μm. **n** Z-DOCK of prediction of the trimer complex structure of circMRPS35 fragment (marked stick), H4K5ac (marked blue cartoon), and KAT7 (marked green cartoon). The significant binding interaction was marked by a red line. **o** ChIRP assay for the binding of circMRPS35 to FOXO1 and FOXO3a promoters in MGC803 cells. LacZ was a negative control. The PCR primers covered P2 (− 1865~ − 2045) in FOXO1 promoter and p1 (− 1560~ − 1739) in FOXO3a promoter, respectively. **p**-**q** ChIP assay for H4K5ac levels in FOXO1 **p** and FOXO3a **q** promoters after KAT7 knockdown. SGC7901 cells were transfected with circMRPS35 or control plasmids, together with KAT7 siRNA and the corresponding control. Cells were harvested for ChIP assay 48 h later. **r** qRT-PCR for FOXO1 and FOXO3a mRNA expression in SGC7901 cells treated as in **p**. **s** Western blot assay for KAT7, FOXO1 and FOXO3a protein levels in cells treated as in **p**. Cells were harvested 48 h later for Western blot analysis. *P < 0.05, **P < 0.01, ***P < 0.001

To further investigate the mechanism of circMRPS35-activated of FOXO1 and FOXO3a transcription, MGC803 lysates were incubated with biotinylated circMRPS35 probe followed by RNA pull-down assay and mass spectrometry (Fig. 7a). The candidate proteins those may likely participate in transcription regulation, including basic transcription factor 3 (BTF3), lysine acetyltransferase 7 (KAT7), and TATA-box binding protein associated factor 15 (TAF15), were evaluated in subsequent validation assays. RIP and RNA pull-down further confirmed that only KAT7 bound specifically to circMRPS35 (Fig. 7b and c). In addition, FISH and immunofluorescence showed that circMRPS35 colocalized with KAT7 in the nucleus of MGC803 cells (Fig. 7d). Molecule docking indicated that circMRPS35 fragment (back-splice sites) formed multiple hydrogen bond interactions with KAT7 residues, including Trp-350, Asp-430, Arg-483, Gly-485, Tyr-486, Gly-487, Lys-488, Lys-524, and Ser-592 (Fig. 7e, Additional file 8: SFig 4a, b and Additional file 9). Subsequently, different mutations of FLAG-tagged KAT7 plasmids were designed for analysis of the interaction between KAT7 and circMRPS35 (Fig. 7f). RNA pull-down assay clearly confirmed that the KAT7 (256-315aa) C2H2 domain was essential for its interaction with circMRPS35 (Fig. 7g). Previous research showed that KAT7 prefers to acetylate H4 at lysine 5, lysine 12 and H3 at lysine 14 [29, 30]. Interestingly, recent studies on patterns of histone acetylation in human genome indicated the potential enrichment of H4K5ac, H4K12ac and H3K14ac in the promoter regions of FOXO1 and FOXO3a (Additional file 8: SFig 4c and d) [31]. Subsequently, ChIP demonstrated that only H4K5ac, neither H4K12ac nor H3K14ac, directly bound to the promoter regions of FOXO1 and FOXO3a in MGC803 cells (Fig. 7h, i and Additional file 8: SFig 4e, f). Figure 7j illustrates that circMRPS35 and H4K5ac were colocalized in the nucleus of MGC803 cells. RNA pull-down and RIP demonstrated that H4K5ac specifically interacted with circMRPS35 (Fig. 7k and l). Furthermore, the colocalization of circMRPS35, KAT7 and H4K5ac was confirmed by super-high-resolution imaging (Fig. 7m), demonstrating that circMRPS35 interacted with both H4K5ac and KAT7 to form a trimer complex. ZDOCK revealed that, in the complex of circMRPS35/KAT7/H4K5ac, residues 77–84 of H4K5ac were docked into the pocket of KAT7 formed by residues 534–540 and residues 563–576 with a β-sheet motif. Moreover, there is an obvious interaction between residues 54–77 with an α-helix motif of H4K5ac and residues 563–576 of KAT7 (Fig. 7n, Additional file 8: SFig 4 g, h and Additional file 10). The chromatin isolation by RNA purification (ChIRP) assay showed that circMRPS35 directly bound to the FOXO1 and FOXO3a promoter regions (Fig. 7o). These combined evidence strongly suggested that circMRPS35 act as a modular scaffold to recruit KAT7 to the promoters of FOXO1 and FOXO3a gene. Additionally, KAT7 knockdown attenuated circMRPS35-increased H4K5ac level in FOXO1 and FOXO3a gene promoters (Fig. 7p and q). Eventually, knockdown of KAT7 significantly reduced circMRPS35-induced FOXO1 and FOXO3a mRNA and protein expression (Fig. 7r and s). In conclusion, circMRPS35 increases the acetylation level of H4K5 in the promoter regions of FOXO1 and FOXO3a through the recruitment of KAT7.

## Discussion

**Fig. 8:**
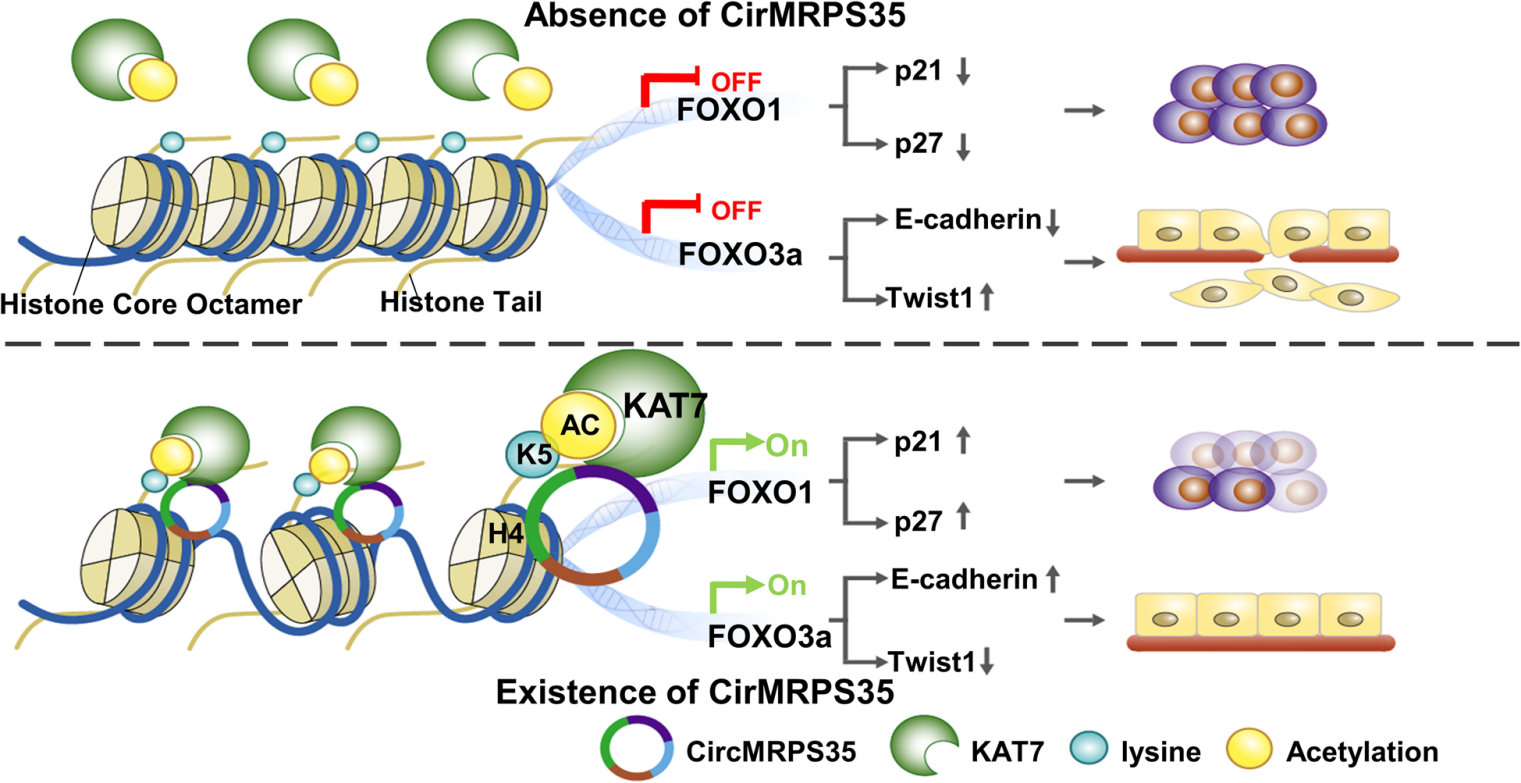
A Schematic Model of CircMRPS35 in the Proliferation and Metastasis of Gastric Cancer. In this model, circMRPS35 alters the histone modification pattern via recruiting KAT7 to FOXO1 and FOXO3a gene promoter regions as a modular scaffold, thereby increasing H4K5 acetylation in their promoter regions, which ultimately alters the downstream genes p21, p27, Twist1 and E-cadherin expression and suppresses the proliferation and invasion of gastric cancer cells

In this study, we identified a novel circRNA named circMRPS35 with an important biological impact on human gastric cancer. The characteristics of circMRPS35 were confirmed through RNase R treatment, Actinomycin D treatment and Sanger sequencing. The expression of circMRPS35 is significantly decreased in gastric cancer tissues compared to the expression in control tissues and is correlated with the clinical characteristics of gastric cancer patients. Overexpression of circMRPS35 significantly inhibits the proliferation and invasion of gastric cancer cells in vivo and in vitro. CircMRPS35 increases H4K5 acetylation associated with the promoter regions of FOXO1 and FOXO3a via recruitment of KAT7, which eventually leads to the upregulation of FOXO1 and FOXO3a (Fig. 8). Altogether, our study not only provides the pivotal roles of circMRPS35 in governing histone modification to activate FOXO1/3a pathways, but also reveals circMRPS35 as a promising diagnostic marker and therapeutic target to combat gastric cancer.

It has been clearly demonstrated that circRNAs may function as miRNA sponges to regulate the expression of target genes, alter the subcellular location of proteins and act as scaffolds to modulate protein-protein interactions. For example, ciRS-7/CDR1as contains more than 70 miR-7 binding sites and then relieves the suppression of miR-7 on CDR1 [18]. In this study, we demonstrated that circMRPS35 participated in chromatin remodeling and increased H4K5 acetylation associated with target genes promoters, which reveals a novel mechanism of circRNA. Chromatin remodeling is the dynamic modification of chromatin structure to allow regulatory elements access to condensed genomic DNA, thus altering gene expression [32]. Studies have mainly focused on the role of protein complexes in chromatin remodeling, including histone modifying enzymes, DNA modifying enzymes and ATP-dependent chromatin remodeling complexes [33, 34]. Interestingly, recent research has revealed that long noncoding RNAs (lncRNAs), such as lncRNA HOTAIR and GClnc1, directly participate in histone modification and modulate target genes transcription [22, 35]. To our knowledge, we are the first to demonstrate that circRNAs specify the histone modification pattern on target genes.

Histone modifications include methylation, acetylation, phosphorylation, ubiquitylation and sumoylation [36]. Histone acetylation is dynamically regulated by histone acetyltransferases (HAT) and histone deacetylases (HDAC). HATs are categorized into at least four families, including the Gcn5/PCAF family, MYST family, p300/CBP family and Rtt109 family. Lysine acetyltransferase 7 (KAT7), also known as HBO1 and MYST2, is a member of the MYST family [29]. KAT7 prefers to acetylate H4 at lysine 5, lysine 12 and H3 at lysine 14. Here we show that, circMRPS35, a new scaffold partner of KAT7, specifically increased the H4K5 acetylation levels in target gene promoter regions. We found for the first time that circRNAs directly affect the acetylation of the regulatory region of target genes and “unlock” their expression, which suggests that noncoding RNAs play a significant role in the histone modification of gene promoters.

FOXO1 and FOXO3a belong to the forkhead box O (FOXO) family of transcription factors. FOXO1 mediates growth arrest and cell apoptosis by activating the transcription of its downstream genes p21, p27, PUMA and Bim [26]. FOXO3a suppresses cell invasion and metastasis through positive regulation of E-cadherin and negative regulation of Twist1 [27]. STAT3 [37] and FOXA2 [38] can directly bind to the promoter of FOXO1 and positively regulate its expression. Many transcriptional factors, such as FOXK2 [39], glucocorticoid receptor (GR) [40], TEA domain family member 1 (TEAD1) [41], STAT3 [37] and AP1 [42], increase the transcription activity of FOXO3a. Our study demonstrates that KAT7 promotes the transcription of FOXO1 and FOXO3a through increased levels of acetylation, which may allow better access to FOXO1 and FOXO3a promoters for the transcriptional factors. As both of FOXO1 and FOXO3a can be augmented by circMRPS35, we speculate that the circMRPS35 itself may be a better therapeutic target. At present, it has been reported that overexpression of native protective circRNAs can be achieved by lentiviral or adenoviral vectors in vivo. They will encode mini-gene cassettes with exon(s), as well as the endogenous splice donor and acceptor sites, and flanking intronic inverted repeats that support RNA backfolding [43]. For instance, microinjection of circDLGAP4 lentivirus significantly ameliorates ischemic stroke outcomes [44]. Moreover, the delivery of circRNAs follows the existing methods for delivering therapeutic linear RNAs, as no specialized possibilities or physicochemical obstructions have yet been found associated with circularity. The strategies contain systemic injection into the vasculature, subcutaneous injection or depots, or local application [43].

In conclusion, our study demonstrates that circMRPS35 recruits histone acetyltransferase KAT7 as a modular scaffold, which subsequently increases the level of H4K5ac in the promoter regions of FOXO1 and FOXO3a and ultimately suppresses the proliferation and invasion of gastric cancer cells. CircMRPS35 expression is positively associated with FOXO1 and FOXO3a expression in patients with gastric cancer. Taken together, our study is the first to demonstrate that circMRPS35 and its associated pathway might be a crucial target for the diagnosis and treatment of gastric cancer.

## Supplementary information

**Additional file 1: Table S1.** Clinicopathological data of 160 gastric cancer patients

**Additional file 2: Table S2.** The list of primers and probes

**Additional file 3: Table S3.** All the identified circRNAs contained at least two independent back-spliced reads in at least two samples in this study

**Additional file 4: Table S4.** The novel circRNAs identified in this study

**Additional file 5: Table S5.** The length distribution of the identified exonic circRNAs

**Additional file 6: Figure S1.** The Validation of Differentially Expressed CircRNAs in 30 pairs of Gastric Cancer Tissues and the Paracancer Tissues

**Additional file 7: Figure S2.** CircMRPS35 Knockdown Promotes MKN45 Cells Proliferation and Metastasis in vitro. Related to Fig. 3

**Additional file 8: Figure S3.** Restoration of FOXO1 and FOXO3a Partially Reversed CircMRPS35-Induced Suppression of Gastric Cancer Progression. Related to Fig. 5. **Figure S4**. Molecular Docking of the Interactions of CircMRPS35/KAT7/H4K5ac and the Design of ChIP Primers. Related to Fig. 7

**Additional file 9: PDB file 1.** The molecular docking of the interaction between circMRPS35 fragment and KAT7

**Additional file 10: PDB file 2.** Z-DOCK of prediction of the trimer complex structure of circMRPS35 fragment, H4K5ac and KAT7

**Additional file 11:** Animal Ethic Statement

## Data Availability

The datasets supporting the conclusions of this article are included within the article and its additional files. The RNA-seq data can be accessed by GEO series accession number GSE121445 (https://www.ncbi.nlm.nih.gov/geo/query/acc.cgi?acc=GSE121445) and GSE121382 (https://www.ncbi.nlm.nih.gov/geo/query/acc.cgi?acc=GSE121382). The data can also be accessed on the UCSC Genome Browser displaying the uploaded sequence tracks (http://genome.ucsc.edu/s/jieMM/hg38_FINAL).

## Abbreviations

ChIP: Chromatin immunoprecipitation
FISH: Fluorescent in situ hybridization
IF: Immunofluorescent staining
IHC: Immunohistochemical staining
RIP: RNA binding protein immunoprecipitation
ROC: Receiver operating characteristic
