# Supplementary material for: CircMRPS35 suppresses gastric cancer progression via recruiting KAT7 to govern histone modification
Source: Mol Cancer. 2020 Mar 12;19:56. doi: 10.1186/s12943-020-01160-2 (PMC7066857; doi:10.1186/s12943-020-01160-2)
Supplement: Supplementary file 6 — Additional file 6: Figure S1. The Validation of Differentially Expressed CircRNAs in 30 pairs of Gastric Cancer Tissues and the Paracancer Tissues [file 12943_2020_1160_MOESM6_ESM.docx]

**
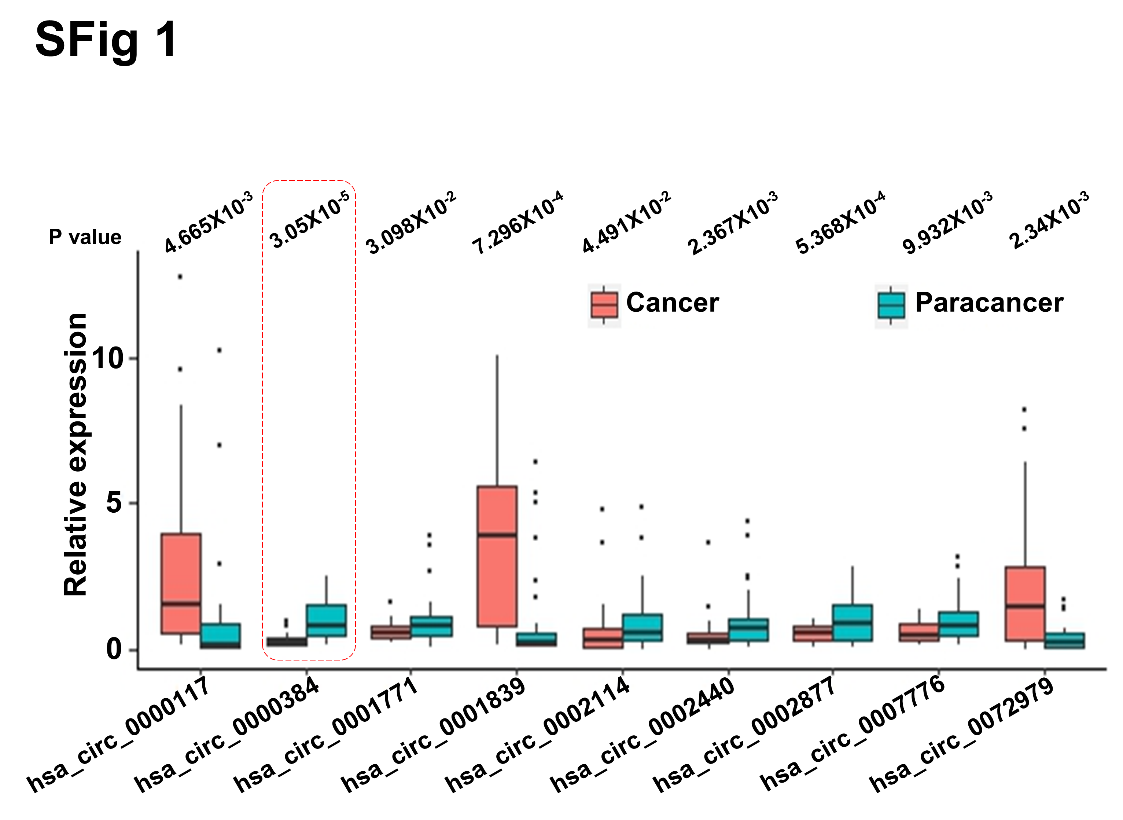
**

**Supplementary Figure 1.** The Validation of Differentially Expressed CircRNAs in 30 pairs of Gastric Cancer Tissues and the Paracancer Tissues. The results were analyzed by ggplot2 and the p value was shown.
