# Supplementary material for: CircMRPS35 suppresses gastric cancer progression via recruiting KAT7 to govern histone modification
Source: Mol Cancer. 2020 Mar 12;19:56. doi: 10.1186/s12943-020-01160-2 (PMC7066857; doi:10.1186/s12943-020-01160-2)
Supplement: Supplementary file 7 — Additional file 7: Figure S2. CircMRPS35 Knockdown Promotes MKN45 Cells Proliferation and Metastasis in vitro. Related to Fig. 3 [file 12943_2020_1160_MOESM7_ESM.docx]

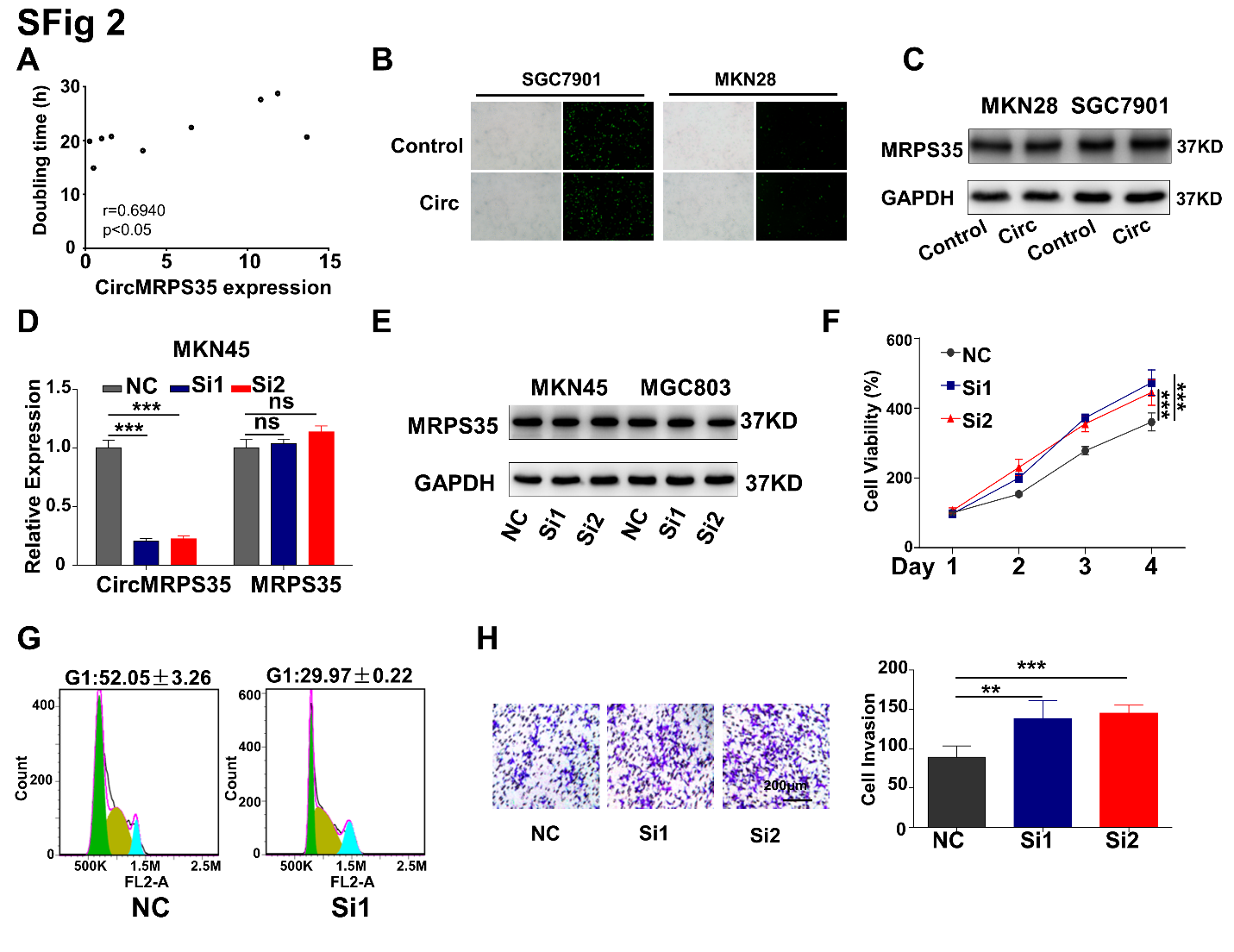


**Supplementary Figure 2. CircMRPS35 Knockdown Promotes MKN45 Cells Proliferation and Metastasis *in vitro*. Related to Figure 3.**

(a) The correlation between circMRPS35 expression and doubling time in gastric cancer cell lines and GES-1 cells. (b) The transfection efficiency of pCDH-CMV-CircMRPS35 and corresponding control plasmids into SGC7901 and MKN28 was observed by fluorescence microscope. (**c**) Western blot for MRPS35 after the transfection of overexpression plasmids into SGC7901 and MKN28 cells for 48 h. (**d**) qRT-PCR assay for circMRPS35 and MRPS35 mRNA in MKN45 cells transfected with two siRNAs targeting circMRPS35. (**e**) Western blot for MRPS35 in MKN45 and MGC803 cells transfected with two siRNAs targeting circMRPS35. (**f**-**h**) CCK-8 (**f**), flow cytometry (**g**) and cell invasion assays (**h**) of the above cells in (**d**). **P<0.01, ***P<0.001.
