# Supplementary material for: CircMRPS35 suppresses gastric cancer progression via recruiting KAT7 to govern histone modification
Source: Mol Cancer. 2020 Mar 12;19:56. doi: 10.1186/s12943-020-01160-2 (PMC7066857; doi:10.1186/s12943-020-01160-2)
Supplement: Supplementary file 8 — Additional file 8: Figure S3. Restoration of FOXO1 and FOXO3a Partially Reversed CircMRPS35-Induced Suppression of Gastric Cancer Progression. Related to Fig. 5. Figure S4. Molecular Docking of the Interactions of CircMRPS35/KAT7/H4K5ac and the Design of ChIP Primers. Related to Fig. 7 [file 12943_2020_1160_MOESM8_ESM.docx]

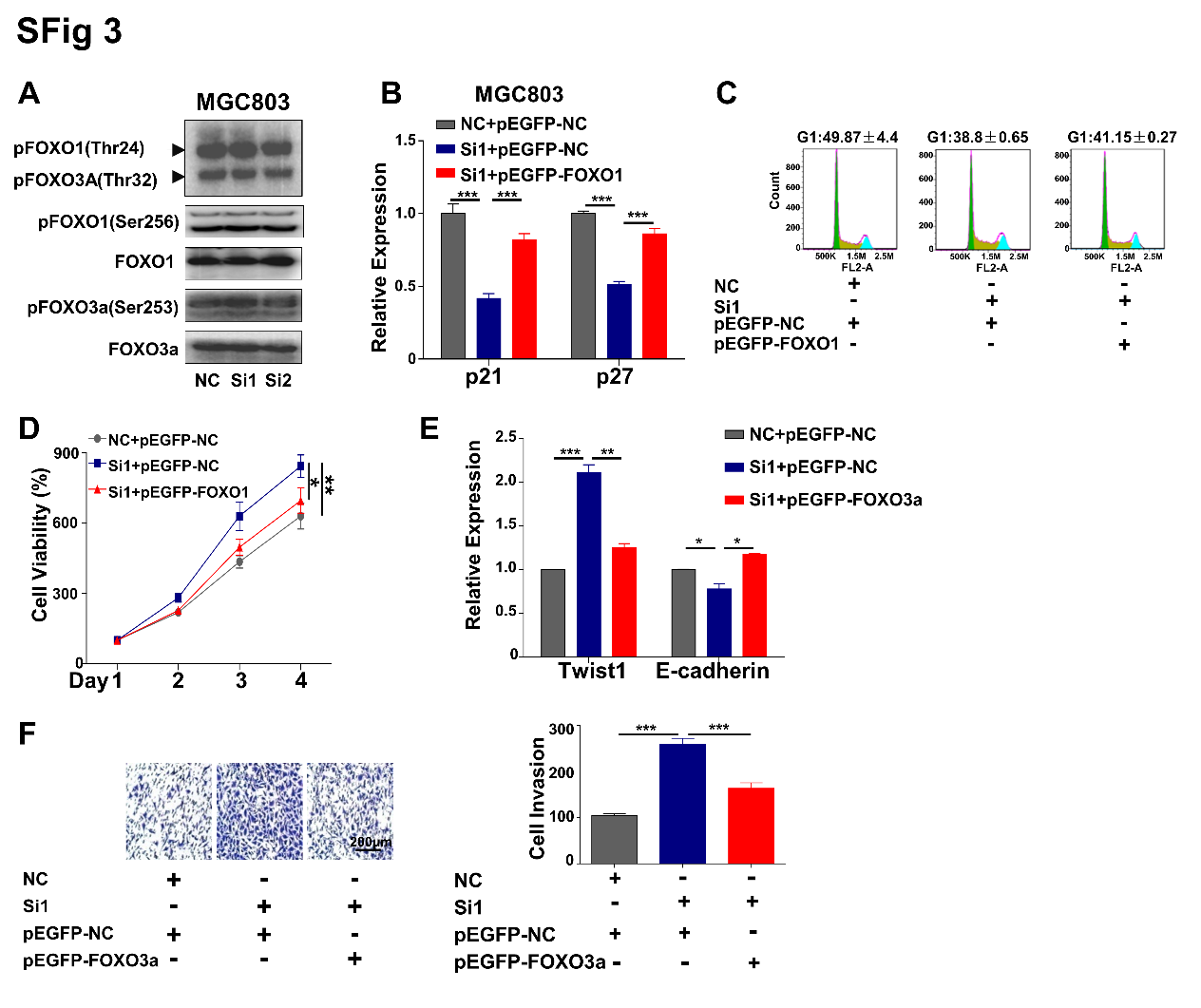


**Supplementary Figure 3. Restoration of FOXO1 and FOXO3a Partially Reversed CircMRPS35-Induced Suppression of Gastric Cancer Progression. Related to Figure 5.**

(**a**) Western blot for p-FOXO1 (Ser256, Thr24) and p-FOXO3a (Ser253, Thr32) after the transfection of two siRNAs targeting circMRPS35 into MGC803 cells for 48 h. Cells were pretreated with actinomycin D (5ug/ml) for 30 min before the transfection. Forty-eight hours later, the phosphorylation of FOXO1 and FOXO3a was measured. (**b**) The mRNA expression of p21 and p27 after restoration of FOXO1. MGC803 cells were transfected with circMRPS35 siRNA (Si1) or corresponding control siRNA (NC), together with pEGFP-NC or pEGFP-FOXO1 plasmids. Forty-eighty hours later, cells were harvested for qRT-PCR analysis. (**c**) Cell cycle was analyzed by flow cytometry. The cells were treated as in (**b**). (**d**) CCK-8 assay was performed in the above cells. The cells were treated as in (**b**) and cell proliferation was tested on the indicated days. (**e**) Twist1 and E-cadherin mRNA expression after restoration of FOXO3a. MGC803 cells were transfected with Si1 or NC, together with pEGFP-NC or pEGFP-FOXO3a plasmids. Forty-eighty hours later, cells were harvested for qRT-PCR analysis. (**f**) Cell invasion assay was performed after restoration of FOXO3a. MGC803 cells were treated as in (**e**), and six hours later, the cells were resuspended and seeded in transwells for another 48 h, and the number of invasion cells was calculated. Scale bar, 200 μm. *P<0.05, **P<0.01, ***P<0.001.

**
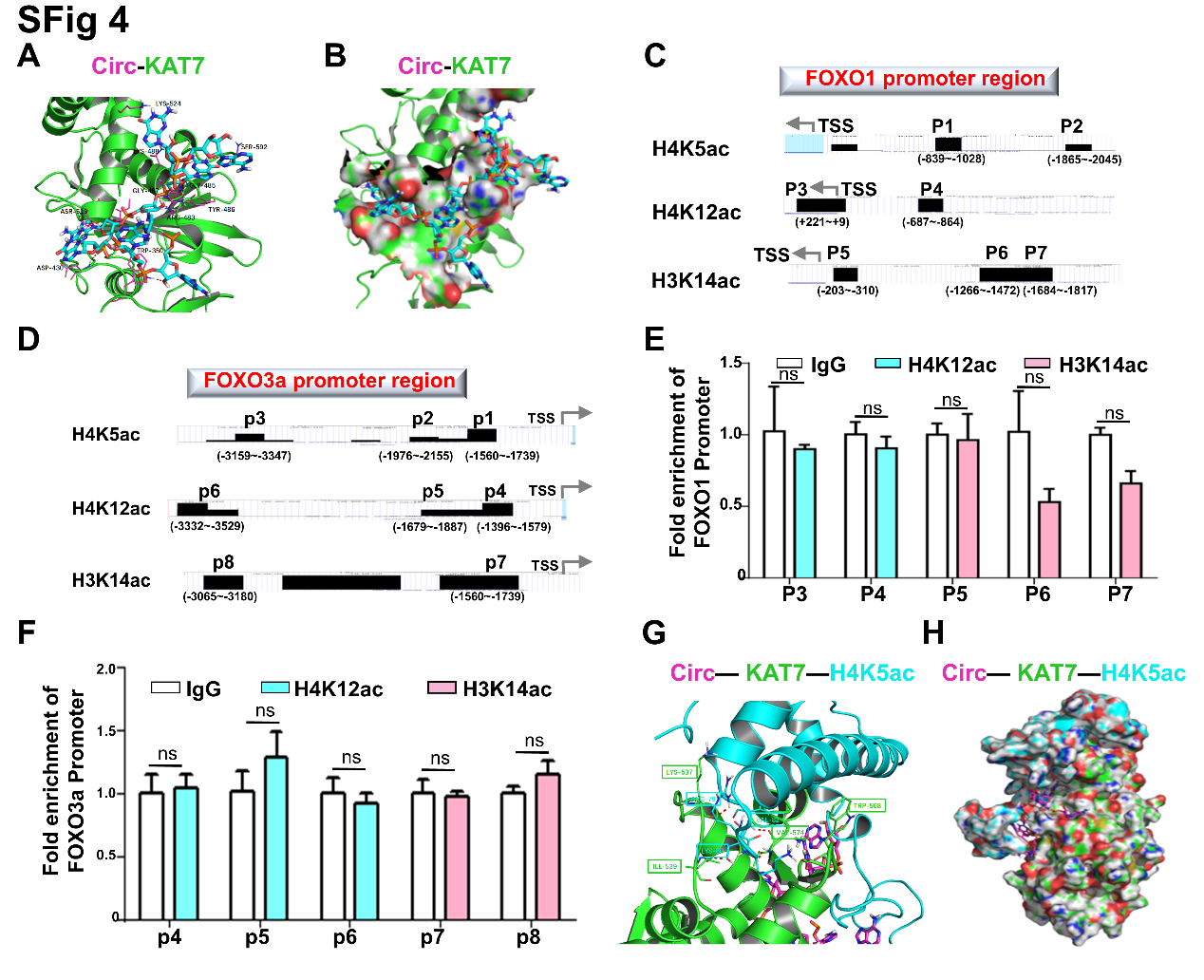
 Supplementary Figure 4. Molecular Docking of the Interactions of CircMRPS35/KAT7/H4K5ac and the Design of ChIP Primers. Related to Figure 7.**

(**a**) The molecular docking of interaction residues between the circMRPS35 fragment (marked stick) and KAT7 (shown as green cartoon). The formed hydrogen bonds are labeled, such as Trp-350, Asp-430, Arg-483, Gly-485, Tyr-486, Gly-487, Lys-488, Lys-524, and Ser-592. (**b**) CircMRPS35 fragment (marked stick) docked in the surface of KAT7. (**c**-**d**) The diagram of the enrichment of H4K5ac, H4K12ac and H3K14ac in the promoter regions of FOXO1 (**c**) and FOXO3a (**d**) revealed by the database (<https://dir.nhlbi.nih.gov/papers/lmi/epigenomes/hgtcellacetylation.aspx>) and design of ChIP primers. (**e**) ChIP assay for H4K12ac and H3K14ac levels in FOXO1 promoter region. PCR primers covered P3 (+9~+221), P4 (-687~-864), P5 (-203~-310), P6 (-1,266~-1,472) and P7 (-1,684~-1,817) in FOXO1 promoter. (**f**) ChIP assay for H4K12ac and H3K14ac levels in FOXO3a promoter region. PCR primers covered p4 (-1,396~-1,579), p5 (-1,679~-1,887), p6 (-3,332~-3,529), p7 (-1,560~-1,739) and p8 (-3,065~-3,180) in FOXO3a promoter. (**g**) CircMRPS35 fragment docked into the surface of KAT7 and H4K5ac revealed by molecular docking. (**h**) Detailed binding interaction of the circMRPS35 fragment (marked stick) with KAT7 (shown as green) and H4K5ac (marked blue) revealed by molecular docking.
