# Supplementary material for: CircMRPS35 suppresses gastric cancer progression via recruiting KAT7 to govern histone modification
Source: Mol Cancer. 2020 Mar 12;19:56. doi: 10.1186/s12943-020-01160-2 (PMC7066857; doi:10.1186/s12943-020-01160-2)
Supplement: Supplementary file 11 — Additional file 11: Animal Ethic Statement [file 12943_2020_1160_MOESM11_ESM.pdf]

动物伦理证明（AMUWEC2019385）

Animal Ethical Statement

兹有第三军医大学胡长江开展的国家自然科学基金Hsa_circ_0000384介导染色质重塑激活FOXO1/FOXO3a转录在抗胃癌中的作用及机制研究课题研究中所使用的动物（裸鼠，性别：雄性，n=150）,符合我国微生物的控制要求。实验设计、实验过程及动物处死方法，经过第三军医大学实验动物福利伦理审查委员会（Laboratory Animal Welfare and Ethics Committee Of the Third Military Medical University）审核，符合动物伦理和动物福利要求。

我单位实验动物生产许可证号为SCXK（渝）20170002，使用许可证号为SYXK（渝）20170002，同意该同志在整个实验的动物操作方法。

中国人民解放军第三军医大学
实验动物福利伦理审查委员会
Laboratory Animal Welfare and Ethics Committee
Of the Third Military Medical University
2019年3月11日
